# Endoplasmic reticulum stress signaling modulates ischemia/reperfusion injury in the aged heart by regulating mitochondrial maintenance

**DOI:** 10.1186/s10020-024-00869-w

**Published:** 2024-07-23

**Authors:** Ji Zhang, Yuanyuan Zhao, Nianqiao Gong

**Affiliations:** 1 Institute of Organ Transplantation, Tongji Hospital, Tongji Medical College, Huazhong University of Science and Technology, Key Laboratory of Organ Transplantation of Ministry of Education, National Health Commission and Chinese Academy of Medical Sciences, Wuhan, Hubei 430030 P.R. China; 2 Department of Urology, The First Affiliated Hospital of Anhui Medical University, Institute of Urology & Anhui Province Key Laboratory of Genitourinary Diseases, Anhui Medical University, Hefei, Anhui 230022 P.R. China

**Keywords:** Heart, Ischemia/reperfusion injury, Endoplasmic reticulum stress, Mitochondrial maintenance, Aging

## Abstract

Aging is associated with an increased risk of myocardial ischemia/reperfusion injury (IRI). With an increasing prevalence of cardiovascular diseases such as coronary arteriosclerosis in older people, there has been increasing interest in understanding the mechanisms of myocardial IRI to develop therapeutics that can attenuate its damaging effects. Previous studies identified that abnormal mitochondria, involved in cellar senescence and oxidative stress, are the master subcellular organelle that induces IRI. In addition, endoplasmic reticulum (ER) stress is also associated with IRI. Cellular adaptation to ER stress is achieved by the activation of ER molecular chaperones and folding enzymes, which provide an important link between ER stress and oxidative stress gene programs. In this review, we outline how these ER stress-related molecules affect myocardial IRI via the crosstalk of ER stress and mitochondrial homeostasis and discuss how these may offer promising novel therapeutic targets and strategies against age-related cardiovascular diseases.

## Introduction

**Fig. 1:**
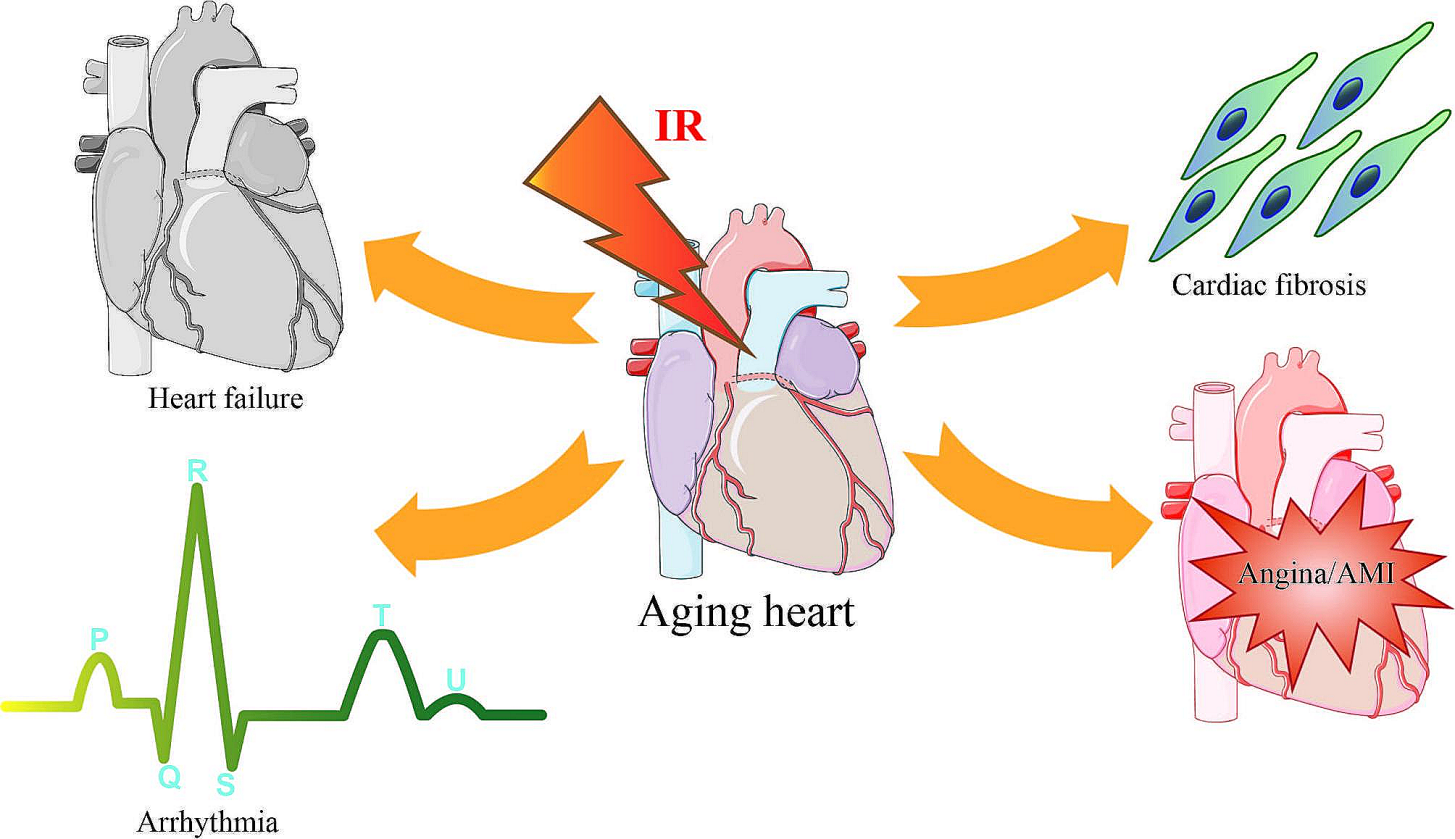
IRI is more likely to occur in the aging heart afflicted with ischemic cardiomyopathy (ICM), such as angina, AMI, arrhythmia, myocardial fibrosis, and heart failure, which are significant causes of cardiovascular disease-related morbidity and mortality

Coronary revascularization procedures and medical therapies have reduced the global age-standardized prevalence, disability-adjusted life years (DALYs), and mortality from acute myocardial infarction (AMI) and coronary artery disease. However, ischemic heart disease remains a major public health challenge. From 1990 to 2019, the global prevalence of ischemic heart disease peaked in the 85–89 group for males and in the 90–94 age range for females (Safiri et al. 2022). Compared to healthy hearts, aged hearts are more susceptible to ischemic insults and undergo greater damage in response to ischemia/reperfusion (IR) (Zhang et al. 2020a, b). They also gain less protection from ischemic preconditioning against ischemia/reperfusion injury (IRI) (Munckhof et al. 2013). A combination of an aging population combined with the fact that individuals aged over 65 years account for over 80% of ischemic heart disease (Rosamond et al. 2007) has led IRI to become the major cause of global cardiovascular disease-related disability and mortality. IRI is characterized by microvascular perfusion defects, platelet activation, and sequential cardiomyocyte death due to additional ischemic events at the reperfusion stage (Wang et al. 2020a, b, c; Obas and Vasan 1979) (Fig. 1). As such, there is a critical need to explore the structural and functional changes of the aging heart during IRI.

**Fig. 2:**
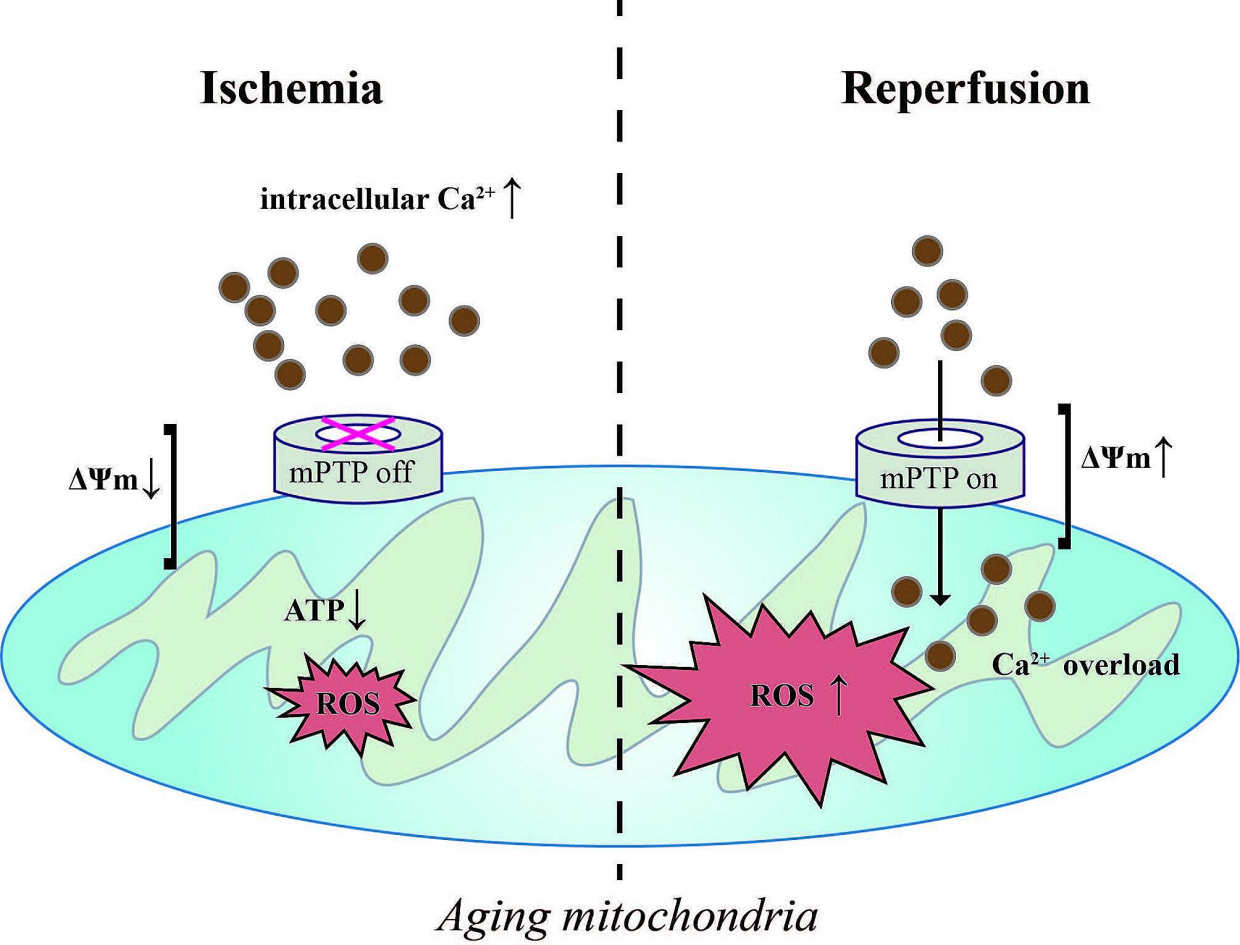
With age, ΔΨm dissipation, loss of ATP production, increasing intracellular Ca^2+^, toxic ROS generated during ischemia gradually disrupt mitochondrial homeostasis. Subsequent reperfusion opens mPTP, eliciting ΔΨm rising, calcium overload, and overproduction of ROS, thereby contributing to mitochondrial damage and, ultimately, cell death

Mitochondria are bioenergetic, biosynthetic, signaling organelles that provide a critical stress-sensing function that helps cells adapt to their environment (Vyas et al. 2016). Over the past few decades, many investigators, including our team, have firmly concluded that the mitochondrion is a central contributor to IRI (Bugger and Pfeil 2020; Wang et al. 2021; Zhang et al. 2021a, b; Kuang et al. 2023). Under ischemic conditions, mitochondrial membrane potential (ΔΨm) is markedly reduced and adenosine triphosphate (ATP) stores become exhausted; this is accompanied by acidosis that is secondary to lactate accumulation, and an increase in the intracellular calcium (Ca^2+^) concentration. Meanwhile, the outer mitochondrial membrane (OMM) remains intact and the mitochondrial permeability transition pore (mPTP) remains closed. With subsequent reperfusion, the reintroduction of oxygen leads to a rapid normalization of pH and a rapid restoration in ΔΨm, precipitating a range of adverse sequelae including the production of mitochondrial reactive oxygen species (ROS), exacerbating Ca^2+^ overload, OMM destruction, and mPTP formation (Kulek et al. 2020). Aging aggravates these mitochondrial events that are initiated by IRI (Campbell et al. 2019; Salehpour et al. 2019) (Fig. 2). Most of the cardiomyocyte oxidative damage can be attributed to these mitochondrial dysfunctions (Paradies et al. 2018). In addition, IR stress also induces mitochondrial biosynthesis, mitochondrial dynamics (fusion-fission)/mitophagy, and mitochondrial proteostasis which facilitate mitochondrial quality control (MQC). It is thought that proper MQC is a compensatory molecular mechanism that removes anomalous mitochondrial proteins or entirely injured mitochondria, and restores mitochondrial function and homeostasis in the setting of IR, favoring cardioprotection (Wang and Zhou 2020). In contrast, MOC disruptions accelerate lethal myocardial IRI (Zhu et al. 2021). Aging impairs mitochondrial function, exacerbating mitochondrial dysfunction and imbalances in MQC, thus leading to greater cardiac injury during IR (Chen et al. 2021).

**Fig. 3:**
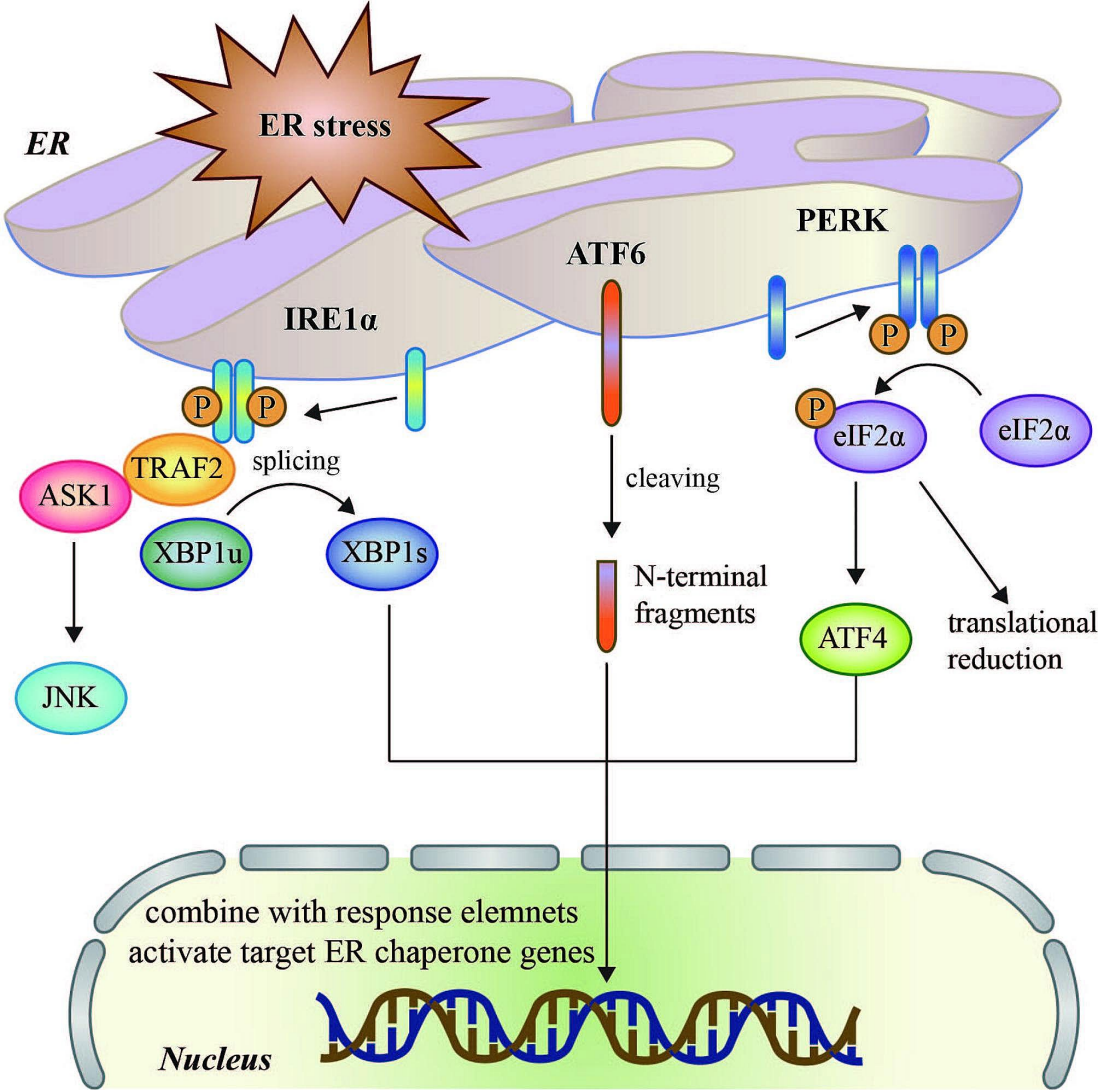
Three response pathways (IRE1α, ATF6, and PERK) of UPR regulate the ER stress response. Upon dimerization and autophosphorylation, IRE1α, a transmembrane RNase, splices XBP1 pre-mRNA to XBP1 mRNA, which is translated into an active transcriptional factor, activating the transcription of UPR genes encoding ER chaperones. IRE1α can also recruit TRAF2 and ASK1, leading to the downstream activation of JNK. The transcription factor ATF6 is synthesized as a transmembrane protein and activated by proteolysis in response to ER stress. Active PERK phosphorylates eIF2α, inhibiting general protein translation. Phosphorylation of eIF2α also allows for the preferential translation of ATF4. Diversified ER chaperone genes are the transcription targets of ATF4

In addition to the mitochondria and oxidative stress, the endoplasmic reticulum (ER) also plays an important role in IR. ER is a dynamic and stable organelle that assists in protein synthesis, modification, and topology, as well as regulating cellular signal transduction mainly via its function as a dynamic Ca^2+^ storage compartment (Görlach et al. 2006). IR can alter ER function, triggering the accumulation of unfolded/misfolded proteins and resulting in evolutionarily conserved cell stress responses, namely ER stress (Zhang et al. 2021a, b). ER stress is marked by glucose-regulated protein 78/immunoglobulin heavy chain-binding protein(GRP78/BiP) and C/EBP homologous protein (CHOP) and activates the unfolded protein response (UPR), which involves three principal ER stress sensors: inositol-requiring enzyme 1α (IRE1α), protein kinase R-like ER kinase (PERK), and activating transcription factor 6 (ATF6) (Zhang et al. 2021a, b; He et al. 2018; Sun et al. 2022). These degrade and remove aberrant proteins from the ER lumen (Zhang et al. 2021a, b; He et al. 2018; Sun et al. 2022) (Fig. 3). The resulting ER stress retards protein translation, increases ER chaperone production, and enhances ER-associated protein degradation (ERAD), which together act to restore intracellular homeostasis (Wang et al. 2014). We previously investigated the effects and mechanisms of ER stress concerning the amelioration of IRI (Zhang et al. 2021a, b), finding that well-organized ER stress helps sustain the viability of cardiomyocytes and the integrity of cardiac function. In contrast, aging increases the risk of excessively activated ER stress, which is closely linked to the pathogenesis of cardiac IRI through mechanisms such as calcium imbalance, free radical overproduction, and ER-dependent cardiomyocyte apoptosis (Zhong et al. 2018; Zhu et al. 2018). However, the role of ER stress in aging-associated cardiac IRI remains incompletely understood.

Because ER stress affects the synthesis and modification of nuclear, cytoplasmic, and mitochondrial proteins, it inevitably modulates mitochondrial function and structure via the UPR signaling pathway during IRI (Zhang et al. 2021a, b). Moreover, mitochondria and ER are physically connected through an inner membrane network, called mitochondrial associated membranes (MAMs), which plays a key role in the interaction between Ca^2+^ communication, mitochondrial dynamics, and ER stress (Tilokani et al. 2018). For example, the inositol 1,4,5-trisphosphate receptor 1/GRP75/voltage-dependent anion channel 1 (IP3R1/GRP75/VDAC1) complex on the MAM is the main channel for calcium transfer from the endoplasmic reticulum to the mitochondria, viz. mPTP (Paillard et al. 2013; Yuan et al. 2022). Since aging increases in ER stress precede mitochondrial dysfunction (Barbero-Camps et al. 2014; Chen et al. 2020), it is now generally accepted that ER stress is a critical trigger of cardiac mitochondrial instability in elderly individuals undergoing IRI (Chen et al. 2020).

The importance of ER stress in the transformation of mitochondrial structure and metabolism, especially in aging and severe stimulation of IR, is reflected by the plethora of studies into ER stress and the associated regulation of mitochondrial maintenance in cardiac IRI (Zhu and Zhou 2021; Yan et al. 2019; Petrovski et al. 2011). In this review, we outline the key molecular pathways and cellular processes initiated by ER stress, and their roles in the mitochondrial modulation of cardiac IRI to identify potential therapeutic targets for cardiac IRI in the aged heart.

## IRE1α

Upon ER stress, IRE1α is auto-phosphorylated and transformed into an active dimer, catalyzing the excision of an intron from the X box-binding protein 1-unspliced isoform (XBP1u) mRNA (Zhang et al. 2021a, b; Sun et al. 2022). The resulting spliced form of XBP1 (XBP1s) is a highly potent transcription factor, which promotes the transcription of target ER chaperones and molecules that regulate mitochondrial maintenance (Wang et al. 2014; Yan et al. 2019; Matsui et al. 2008; Bi et al. 2018; Zhang et al. 2023). The level of XBP1 is significantly increased in the aging heart; XBP1s is considered an anti-aging factor with the ability to prevent or delay heart failure (Duan et al. 2016; Jiao et al. 2012). During cardiac IR, IRE1α, XBP1s, GRP78/BiP, and CHOP are increased in hypoxic or reoxygenated cardiomyocytes at the mRNA and protein levels, suggesting that activation of the IRE1α-XBP1s axis is the most highly conserved branch in ER stress (Liu and Dudley 2014).

XBP plays a dual role in that it aggravates and defends against IRI. Several studies from our laboratory and others have revealed that IRE1α-XBP1 induces the transcriptional upregulation of hydroxymethylglutaryl reductase degradation 1 (HRD1) (Zhang et al. 2021a, b; Cominacini et al. 2015; Wu et al. 2014). HRD1, also called synoviolin 1 (SYVN1), is an E3 ubiquitin ligase coordinating ERAD that directly catalyzes ubiquitin conjugation onto unfolded or misfolded proteins for proteasomal degradation (Binder et al. 2022). We already know that HRD1 can downregulate nuclear factor erythroid 2-related factor 2 (NRF2 coded by nuclear factor, erythroid 2 like 2 (NFE2L2) through a ubiquitination-degradation pathway, while HRD1 inhibition can prevent the downregulation of NRF2 in cardiomyocytes (Zhang et al. 2021a, b; Wu et al. 2014; Binder et al. 2022). Importantly, the transcription factor NRF2 is best known as an anti-aging element (Cominacini et al. 2015; Zhang et al. 2020a, b), reducing myocardial infarct size and cardiac hypertrophy in the transition to heart failure in animals exposed to IRI (Cominacini et al. 2015). In terms of interaction with mitochondria, NRF2 regulates responses to mitochondrial-derived ROS via its effects on antioxidant and detoxification genes, including NAD(P)H: quinone oxidoreductase (NQO1), heme oxygenase-1 (HO-1), and superoxide dismutase-1 (SOD-1) (Cominacini et al. 2015; Zhang et al. 2020a, b; Chen and Maltagliati 2018). NRF2 transcription can also increase mitochondrial biogenesis through the expression of genes that are essential for mitochondrial biogenesis such as nuclear respiratory factor 1 (Athale et al. 2012; Chen 2021).

In addition, XBP1 couples ER stress with the hexosamine biosynthetic pathway (HBP) by triggering HBP activation of the major HBP enzymes, glutamine fructose-6-phosphate aminotransferase 1 (GFAT1), glucosamine-phosphate N-acetyltransferase (GNPNAT1), phosphoglucomutase 3 (PGM3), and uridine diphosphate-glucose 4-epimease (GalE) (Wang et al. 2014). The IRE1α-XBP1s-HBP axis promotes the synthesis of uridine diphosphate N-acetylglucosamine (UDP-GlcNAc), which is the obligate substrate for O-linked GlcNAc modification (O-GlcNAcylation) on cardiac proteins under IRI conditions (Wang et al. 2014). O-GlcNAcylation, a prosurvival pathway that counterbalances the age-related decline in self-healing capacity (Jiang et al. 2017), can modulate protein stability and function and has been implicated in various IRI-related cardiovascular diseases. Current evidence suggests that increasing O-GlcNAcylation during IRI may represent a unique endogenously recruitable mechanism of cardioprotection via directly O-GlcNAcylating VDAC in mitochondria (Ngoh et al. 2008, 2009; Jones et al. 2008). Because VDAC is a putative member of the mPTP, O-GlcNAcylation of VDAC prevents the formation of the mPTP, alters sensitivity to the loss of ΔΨm, relieves Ca^2+^ overload-induced mitochondrial swelling, and hence maintains mitochondrial stability (Ngoh et al. 2008).

XBP1 is not the only RNA targeted by the RNase activity of IRE1α. Prolonged ER stress attenuates the expression of XBP1, but continues to engage regulated IRE1-dependent decay (RIDD) and IRE1α-stimulation of the apoptotic-signaling kinase-1 (ASK1) (Nishitoh et al. 2002), which causes activation of Jun-N-terminal kinase (JNK) (Urano et al. 2000). ASK1 participates in the regulation of NRF2 phosphorylation and dissociation from Kelch-like ECH-associated protein 1 (Keap1) (Nishitoh et al. 2002), leading to the nuclear translocation of NRF2. JNK resists cardiac IRI by repressing the mitochondrial fission factor (Mff)-mediated mitochondrial fission and the Bcl-2/adenovirus E1B 19 kDa interacting protein-3 (Bnip-3)-required mitophagy (Jin et al. 2018).

## ATF6

ATF6 has two isoforms, ATF6α (90 kDa) and ATF6β (110 kDa), and is a transmembrane sensor protein that becomes a powerful transcription factor upon regulated intramembrane proteolysis-specific cleavage in the Golgi complex (Liu and Dudley 2014). After cleavage, the cytosolic N-terminal fragments of ATF6, containing conserved DNA-binding domains and divergent transcriptional activation domains, translocate into the nucleus, where it binds to several types of regulatory sequences in target ER stress element (ERSE) genes, and activates IRI-mediated ER stress response gene transcription (Tadimalla et al. 2008). The ATF6α arm of ER stress safeguards organelle homeostasis and cellular aging (Wang et al. 2018); it can be activated in ischemia and inactivated upon reperfusion (Liu and Dudley 2014), serving protective roles in the IRI heart through its induction of numerous ER stress-related molecules, especially following ischemic preconditioning (Brooks et al. 2014).

During IR, ATF6 was identified to underlie the altered expression of 18 genes. Twelve oxidative stress response genes are upregulated by ATF6; they encode proteins with strong antioxidant and scavenging effects on free radicals. Six genes that respond to oxidative stress are decreased by ATF6 but do not encode antioxidants (Jin et al. 2017; Glembotski et al. 2020; Marsh et al. 2021). The most well-characterized of these in cardiac myocytes subjected to IR include peroxiredoxin 5 (Prdx5), catalase (CAT), valosin-containing protein-interacting membrane seprotein (Vimp), and fatty acyl-CoA reductase 1 (FAR1) (Jin et al. 2017). Prdx5 is a member of the family of mammalian proteins that neutralize ROS and is highly constitutively expressed in mammalian hearts but its basal levels are reduced in aged hearts (Kropotov et al. 2007; Edwards et al. 2003). In cardiomyocytes, Prdx5 is distributed in several cellular locations including the mitochondria, peroxisomes, cytosol, and nucleus; it possesses mitochondria and peroxisome targeting sequences, and plays a major role in mitochondrial antioxidant defenses by catalyzing the reduction of potentially damaging peroxides and superoxide through thioredoxin (Jin et al. 2017; Agborbesong et al. 2023). CAT is the longest-recognized antioxidant enzyme; it plays a key role in the metabolism of hydrogen peroxide (H_2_O_2_) and reactive nitrogen species and is expressed and localized in cardiac peroxisomes, catalase, cytosol, and mitochondria (Jin et al. 2017; Dieterich et al. 2000). A CAT-overexpressing transgene was shown to prolong lifespan and resist cardiac IRI by attenuating ROS levels (Li et al. 1997; Ren et al. 2007). Vimp, an ER-transmembrane protein that is also called selenoprotein S (SelS), is one of the only 25 genes that encodes the 21st amino acid selenocysteine in humans (Schomburg 2011), contributing to selenium-mediated protection from oxidative stress in cardiomyocytes (Jin et al. 2017). Recently, Far1 was demonstrated to be an ATF6-dependent ER stress-inducible gene, which encodes a protein that localizes to peroxisomes of cardiac myocytes during IR (Marsh et al. 2021). Elevated FAR1 induces robust plasmalogen production during the onset of reperfusion and forms plasmalogen catabolism by an excess of ROS generated at the mitochondria during reperfusion. Nevertheless, FAR1 may lead to a lethal accumulation of lysophospholipids and α-hydroxyaldehydes, which impair myocyte viability during IR (Marsh et al. 2021). Given that ATF6-induced antioxidant proteins are present inside proteasomes, and proteasomes have been found to decorate the surface of mitochondria (Jin et al. 2017; Glembotski et al. 2020; Marsh et al. 2021), a molecular mechanism involving ATF6 regulation of mitochondria may involve the neutralization of some of the ROS generated in mitochondria during cardiac IR.

According to our current understanding, the expression of some microRNAs (miRs) is significantly upregulated during myocardial IR and heart failure (Zhao et al. 2022a, b; Hulot and Masurkar 2020). Apart from its ability to transcribe potentially protective genes, ATF6 also regulates miR expression in the mouse heart (Belmont et al. 2012). Recently, a whole-genome miR array analysis of RNA from the hearts of ATF6 transgenic mice was conducted to determine the mechanisms of ATF6-mediated protection (Belmont et al. 2012). ATF6 was found to upregulate miR-721 and miR-130b, which were predicted to target the ATF6-regulated mRNA of mitochondrial uncoupling protein 3 (UCP3). UCP3, which is predominantly expressed in the heart (Bézaire et al. 2007), is essential for the recovery of long-chain fatty acid (LCFA) oxidation, mitochondrial respiratory capacity (MRC), and contractile function after myocardial IR (Edwards et al. 2018). Mice lacking UCP3 have larger infarcts after IR compared to wild-type mice. Adult cardiomyocytes from Ucp3 knockout mice show mitochondrial dysfunction, increased ROS production, and apoptotic cell death when compared with equivalent wild-type cells during in vitro hypoxia (Perrino et al. 2013).

## PERK

IR-stimulated activation of the PERK signaling pathway is important for mitochondrial homeostasis and cell survival, but the mechanisms remain incompletely understood. PERK activation causes the phosphorylation of the α-subunit of eukaryotic initiation factor 2 (eIF2α) that reduces protein translation, thereby allowing the cell to fold proteins that are already present in the ER (Wang et al. 2020a, b, c; Latorre-Muro et al. 2021). The exception is ATF4, which avoids translational attenuation induced by eIF2α phosphorylation, owing to the upstream open reading frames (ORFs) in its 5′-untranslated region. Under normal conditions, the upstream ORFs prevent the translation of true ATF4, but phosphorylated eIF2α neglects these and increases the selective translation of the mRNA encoding transcription factor ATF4 (Zhang et al. 2017). ATF4 is a translational target of activated PERK in cardiovascular diseases. Young tissues possess an efficient PERK-eIF2α activation adaptive mechanism but this declines with aging (Hussain and Ramaiah 2007; Boriushkin et al. 2015).

The integrated stress response (ISR) is a central signaling network that responds to proteostasis defects by tuning the rates of protein synthesis (Costa-Mattioli and Walter 2020). It thereby protects the heart from IRI by inhibiting protein synthesis (Zhang et al. 2021a, b). The PERK-eIF2α cascade is strongly induced by IR in cardiomyocytes in vitro and in vivo and is the cardinal pathophysiological mechanism underlying ISR (Wang et al. 2020a, b, c; Tian et al. 2021; Sun et al. 2019). PERK-eIF2α inhibits the NADH-ubiquinone oxidoreductase complex assembly factor 2 (NDUFAF2), an assembly factor of the mitochondrial complex I, by directly targeting translational suppression and alleviating mitochondrial complex-derived ROS in response to IR (Zhang et al. 2021a, b). Notably, PERK also phosphorylates an unidentified site that lies within the amino terminus of the Neh2 domain of NRF2, which is vital for its interaction with Keap1. It thereby frees NRF2 from its inhibitor and promotes its nuclear translocation to modulate the transcriptional response to preserve cellular homeostasis, in an eIF2α phosphorylation-independent manner (Yamamoto et al. 2018). Concomitantly, PERK can phosphorylate O-GlcNAc transferase (OGT) which, in turn, O-GlcNAcylates the translocase of the mitochondrial import receptor outer mitochondrial membrane 70 (TOM70). This then enhances mitochondrial protein import 19 (MIC19), causes mitochondrial cristae formation, and sustains cellular bioenergetics during conditions of high energy demand to maintain cellular survival and activity (Latorre-Muro et al. 2021; Fahie et al. 2022).

Furthermore, the PERK located at the contact sites between the ER and the mitochondria creates a PERK-mitochondria axis that allows PERK to detect stress in both organelles, adapt their functions, and prevent apoptosis (Almeida et al. 2022). IRI-initiated PERK strengthens MQC by promoting mitophagy, which leads to the renewal of the mitochondrial network (Matsui et al. 2008; Almeida et al. 2022; Kouroku et al. 2007).

### GRP94

GRP94, the only member of the heat shock protein (HSP) 90 family that is present in the ER, is coordinately regulated with the GRP78/BiP chaperone system in protein processing and is inducible by ER stress at the transcriptional level (Santana-Codina et al. 2020; Vitadello et al. 2003; Ni et al. 2011). GRP94 decreases with age leading to a significant decrease in the level of IRE1α-XBP1 and a compensatory upregulation of the ER chaperones GRP78/BiP, calnexin, and calreticulin (Mao et al. 2010; Song et al. 2022). The functions of GRP94 include increasing the folding capacity of the ER, assisting in the targeting of malfolded proteins to ERAD, and storing Ca^2+^ (Eletto et al. 2010). Similar to GRP78/BiP, GRP94 exerts pro-survival functions in cells subjected to ER stress. Following exposure of cells to ER stress induced by IR, a deregulated increase in intracellular Ca^2+^ can activate mitochondrial Ca^2+^ uptake, deteriorating normal mitochondrial function, and increasing the production of mitochondrial-generated ROS (Vitadello et al. 2003; Ni et al. 2011). Accordingly, GRP94 protects cardiomyocytes from calcium overload by counteracting the increase of intracellular Ca^2+^ during IR (Vitadello et al. 2003), but the specific mechanisms still need to be clarified.

## Glycogen synthase kinase-3 (GSK-3β)

GSK-3β, an evolutionarily conserved serine/threonine kinase, regulates numerous signaling transduction systems and pathological states that span across cell growth, inflammation, apoptosis, and heart failure (Sharma et al. 2020). The aged heart shows increased levels of activated GSK-3β compared with hearts from young controls (Zhu et al. 2010). In particular, GSK-3β participates in the apoptosis and necrosis of cardiomyocytes after hypoxia/reoxygenation (H/R) (Juhaszova et al. 2009) and occupies an integration point for ER-derived and mitochondria-derived cell death/survival pathways (Miki et al. 2009; Xie et al. 2016). Many therapeutics have attempted to exploit GSK-3β to treat IRI (Kuang et al. 2023; Sharma et al. 2020). We have determined that methyl eugenol (ME), an analog of the phenolic compound eugenol, binds to the binding pocket of Adenosine 5‘-monophosphate (AMP)-activated protein kinase (AMPK) with high affinity, activating the AMPK/GSK3β axis. This, in turn, blocks the NRF2 nuclear export signal and promotes the transcription of antioxidant target genes that help maintain mitochondrial homeostasis (Kuang et al. 2023). Other therapeutic agents, such as fisetin, were also reported to mitigate myocardial IRI and reduce mitochondrial oxidative stress by inhibiting GSK-3β (Sharma et al. 2020; Shanmugam et al. 2018).

IR elevates ROS levels, mostly within and proximal to mitochondria, and can excite mPTP opening, which directly induces ΔΨm loss, with deleterious consequences for the affected mitochondria (Juhaszova et al. 2009; Sheng et al. 2018). The nonphosphorylated form of GSK-3β is constitutively active and forms a complex with two core subunits of mPTP, adenine nucleotide translocase (ANT) and VDAC, diminishes sensitization of mPTP opening, and exerts cardioprotection after IR (Miki et al. 2009). Unfortunately, studies have shown that ER stress can reverse this protective effect by phosphorylating GSK-3β at Ser9 and reducing its activity and mitochondrial translocation, ultimately augmenting the mPTP opening threshold (Miki et al. 2009; Xie et al. 2016).

ER is regarded as a storage location of intracellular zinc (Zn^2+^) (Ridlo et al. 2021). Interestingly, stimulation of cardiac IR has been confirmed to provoke the loss of intracellular Zn^2+^ resulting in the release of Zn^2+^ from the ER. This disturbs Zn^2+^ homeostasis which normally helps mediate ER stress and plays a role in the protective action of ER stress inhibition of the inactivation of GSK-3β-mediated suppression of mPTP (Sheng et al. 2018; Wang et al. 2016).

## Peroxisome proliferator-activated receptor γ co-activator 1-α (PGC-1α)

PGC-1α belongs to a small family of transcriptional coactivators. It plays a pivotal role as a metabolic sensor, is abundant in oxidative active myocardium, and is responsible for the fine-tuning of transcriptional responses to environmental stimuli, determining mitochondrial composition and content (Ding et al. 2018; Packer 2020). PGC-1α regulates the changes that occur in mitochondria during aging; as such, modulation of PGC-1α may offer a therapeutic target for age-related pathology (Halling et al. 2019). Mice with PGC-1α gene deletion have impaired cardiac reserve under stress conditions, but supraphysiological expression of PGC-1α in the heart also leads to cardiomyopathy and heart failure in mice (Arany et al. 2005; Leone et al. 2005). This U-shaped relationship between expression and function suggests that moderate PGC-1α expression is likely to benefit cardiac IRI.

In the myocardium of diabetic rats subjected to IR, ischemic preconditioning with low-dose ER stress induced the expression of PGC-1α, which regulated mitochondrial biogenesis and mitochondrial energy metabolism by interacting with NRF1/NRF2 and mitochondrial transcription factor A (TFAM) (Chen et al. 2022). Beyond this function, ischemic preconditioning induced PGC-1α-mediated mitochondrial fusion-fission directly by binding to key transcriptional promoters, including mitofusin2 (MFN2), dynamin-related protein 1 (DRP1), phosphatase and tensin homolog (PTEN)-induced putative kinase protein1 (PINK1), and Parkin protein, thereby attenuating IRI (Yan et al. 2019; Chen et al. 2022).

## Hypoxia-inducible factor 1 (HIF-1)

The transcriptional complex HIF-1 is a key element in sensing changes in cellular oxygen tension. It comprises an oxygen-sensitive α-subunit, which can both promote and limit longevity, and a constitutively-expressed β-subunit, which is rapidly degraded under normoxia and stabilized during hypoxic conditions (Leiser and Kaeberlein 2010; Leiser et al. 2011). It has been increasingly recognized that HIF-1 orchestrates protective responses to hypoxia through the transcriptional activation of a series of hypoxia-sensitive genes (Ong et al. 2014). As expected, HIF-1 plays an ascendant role in mediating cardioprotection against IRI (Ong et al. 2014; Fukuda et al. 2007; Semenza 2011; Belaidi et al. 2016; Yang et al. 2016, 2020). Current evidence supports that ER stress is a new myocardial activator of HIF-1 during cardiac IRI (Belaidi et al. 2016).

Under hypoxic conditions, HIF-1 regulates the expression of the cytochrome c oxidase (COX) 4 subunit by activating the transcription of genes encoding COX4-2 and LON, a mitochondrial ATP-dependent protease required for COX4-1 degradation, optimizing the efficiency of mitochondrial respiration and restraining mitochondrial-derived ROS (Fukuda et al. 2007). Additionally, HIF-1 reprograms basal cell metabolism from oxidative phosphorylation (OXPHOS) towards aerobic glycolysis (AG), thus suppressing the production of mitochondrial oxidative stress and mPTP opening during IRI. This occurs via the activation of glycolytic protein, pyruvate dehydrogenase kinase-1 (PDK1), and mitochondrial hexokinase II (HKII) (Ong et al. 2014). Of note, HIF-1 also activates the gene encoding Bnip-3, which triggers selective mitophagy and restores mitochondrial maintenance throughout myocardial IRI (Yang et al. 2020).

## Conclusions and perspectives

**Fig. 4:**
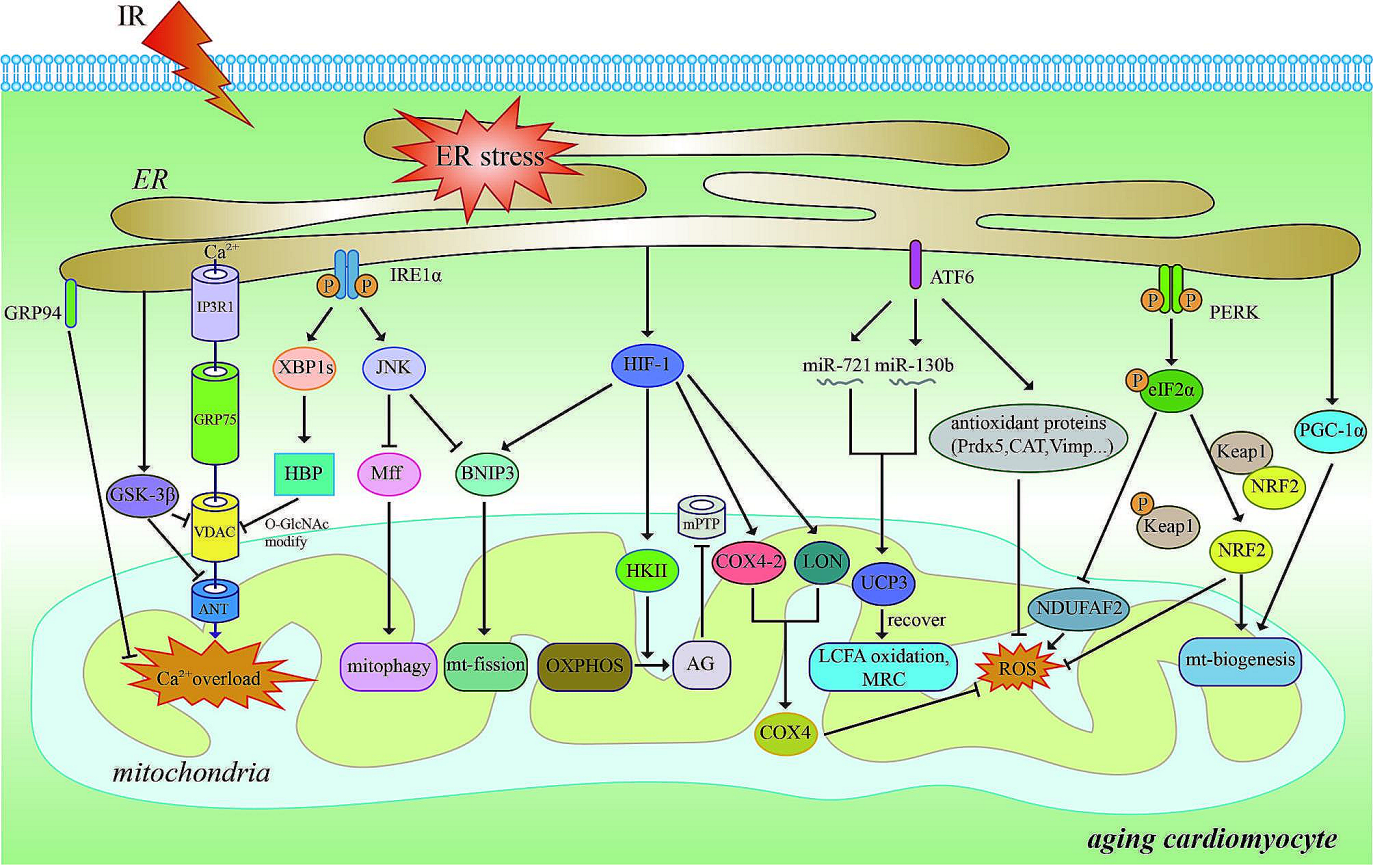
IRE1α, ATF6, PERK, GRP94, GSK-3β, PGC-1α, and HIF-1 are all stimulated by ER stress when the aged heart undergoes IRI. They act on mitochondrial structure and function in cardiac IRI via mechanistically distinct pathways

Our current understanding of myocardial IRI stresses the important influence of ER stress over mitochondrial energy metabolism, Ca^2+^ influx, MQC, and mitochondrial ROS production through diverse molecular pathways (Fig. 4). As the nexus between IRI and mitochondria in the aged heart, the multiple biological processes involved in ER stress are interdependent, complex, highly orchestrated, and even appear to be contradictory. In general, ER stress triggered by mild IR, such as ischemic preconditioning, is beneficial to preserving mitochondrial function and eliminating injured mitochondria. Correspondingly, overwhelming ER stress, which is more likely to appear with aging cardiac IR, is always accompanied by aberrant mitochondrial homeostasis. This phenomenon has been observed in the heart, kidney, liver, pancreatic islets, and other tissues in our previous explorations (Wang et al. 2021; Zhang et al. 2021a, b; Kuang et al. 2023, 2024; He et al. 2018; Gong et al. 2022; Ni et al. 2023). As an example, XBP1s, the principal effector of the ER stress sensor IRE1α, is of particular interest to us. Following XBP1s deficiency, we noted augmentation of forkhead box O1 (FoxO1)-dependent mitophagy in hepatocytes and reduction in caspase-1-dependent mitochondrial damage in renal tubular epithelial cells (Ni et al. 2023; Kuang et al. 2024). This suggests that controlling the extent and form of ER stress may be used to regulate IRI in various organs including the heart. For the elderly with a higher risk of AMI, diabetes mellitus, and other cardiovascular-related diseases, it may be necessary to perturb ER stress or interrupt the crosstalk between the ER and mitochondria (Sheng et al. 2018; Chen et al. 2019; Tan et al. 2020). Current researchers have endeavored to diminish the formation of MAMs by decreasing the mitochondrial-ER proximity or disrupting the IP3R1-GRP75-VDAC1 complex during ER stress, blocking Ca^2+^ communication between the ER and mitochondria, ultimately safeguarding cardiomyocytes from IRI (Li et al. 2024; Zhang et al. 2024; Kirshenbaum et al. 2024). Given the adaptive changes in cardiac IRI and the age-dependent decline of certain UPR molecules and their downstream products in the heart, upregulation of these ER stress pathways has become a target of anti-aging cardiac IRI research (Chen et al. 2020; Edwards et al. 2003; Blackwood et al. 2020). In summary, these evolving findings present encouraging implications for the application of ER-mitochondrial correlations in improving cardiac IRI of aging patients in the clinic.

Admittedly, how IR-induced ER stress modulates mitochondrial maintenance remains incompletely understood. For instance, after testing GSK-3β, PGC-1α, and HIF-1 regulation by ER stress in cardiomyocytes, their regulatory UPR pathway remains unidentified. A number of nascent hypotheses have emerged. It seems that the calcium/calmodulin-dependent protein kinase IIγ (CaMKIIγ) which is capable of pushing mitochondrial Ca^2+^ uptake, loss of ΔΨm, and release of apoptogens may serve as a unifying link between ER stress and mitochondrial apoptotic pathways in irreversible cardiac IRI (Vila-Petroff et al. 2007; Timmins et al. 2009; Feng and Anderson 2017). Besides, despite preclinical studies indicating the potential effectiveness of plentiful ER stress and mitochondrial-targeted therapies in treating myocardial IRI, their clinical implementation still remains insufficient. Because of notable distinctions among different cardiovascular diseases, scholars are compelled to employ distinct animal models tailored to each condition for a more comprehensive exploration of the underlying pathogenic mechanisms of each disease, and a thorough evaluation of the efficacy of novel therapies, while the applicability of animal models to humans also requires further investigation.

## Data Availability

Not applicable.

## Abbreviations

IRI: Ischemia/reperfusion injury
ER: Endoplasmic reticulum
DALYs: Disability-adjusted life years
AMI: Acute myocardial infarction
IR: Ischemia/reperfusion
ΔΨm: Mitochondrial membrane potential
ATP: Adenosine triphosphate
OMM: Outer mitochondrial membrane
mPTP: Mitochondrial permeability transition pore
ROS: Reactive oxygen species
MQC: Mitochondrial quality control
GRP78/BiP: Glucose-regulated protein 78/immunoglobulin heavy chain-binding protein
CHOP: C/EBP homologous protein
UPR: Unfolded protein response
IRE1α: Inositol-requiring enzyme 1α
PERK: Protein kinase R-like ER kinase
ATF6: Activating transcription factor 6
ERAD: ER-associated protein degradation
MAMs: Mitochondrial associated membranes
IP3R1: Inositol 1,4,5-trisphosphate receptor 1
VDAC: Voltage-dependent anion channel
XBP1: X box-binding protein 1
HRD1: Hydroxymethylglutaryl reductase degradation 1
SYVN1: Synoviolin 1
NRF2: Nuclear factor erythroid 2-related factor 2
NQO1: NAD(P)H: quinone oxidoreductase
HO1-: Heme oxygenase-1
SOD1-: Superoxide dismutase-1
HBP: Hexosamine biosynthetic pathway
GFAT1: Glutamine fructose-6-phosphate aminotransferase 1
GNPNAT1: Glucosamine-phosphate N-acetyltransferase
PGM3: Phosphoglucomutase 3
GalE: Uridine diphosphate-glucose 4-epimease
UDP: GlcNAc-Uridine diphosphate N-acetylglucosamine
O: GlcNAcylation-O-linked GlcNAc modification
RIDD: IRE1-dependent decay
ASK1: Apoptotic-signaling kinase-1
JNK: Jun-N-terminal kinase
Keap1: Kelch-like ECH-associated protein 1
Mff: Mitochondrial fission factor
Bnip3-: Bcl-2/adenovirus E1B 19 kDa interacting protein-3
ERSE: ER stress element
Prdx5: Peroxiredoxin 5
CAT: Catalase
Vimp: Valosin-containing protein-interacting membrane seprotein
FAR1: fatty acyl-CoA reductase 1
H_2_O_2_: Hydrogen peroxide
SelS: Selenoprotein S
miRs: MicroRNAs
UCP3: Uncoupling protein 3
LCFA: Long-chain fatty acid
MRC: Mitochondrial respiratory capacity
eIF2α: A-subunit of eukaryotic initiation factor 2
ORFs: Open reading frames
ISR: Integrated stress response
NDUFAF2: NADH-ubiquinone oxidoreductase complex assembly factor 2
OGT: O-GlcNAc transferase
TOM70: Translocase of the mitochondrial import receptor outer mitochondrial membrane 70
MIC19: Mitochondrial protein import 19
HSP: Heat shock protein
GSK-3β: Glycogen synthase kinase-3
H/R: Hypoxia/reoxygenation
ME: Methyl eugenol
AMP: Adenosine 5‘-monophosphate
AMPK: AMP-activated protein kinase
ANT: Adenine nucleotide translocase
PGC-1α: Peroxisome proliferator-activated receptor γ co-activator 1-α
TFAM: Mitochondrial transcription factor A
MFN2: Mitofusin2
DRP1: Dynamin-related protein 1
PTEN: Phosphatase and tensin homolog
PINK1: PTEN-induced putative kinase protein1
HIF-1: Hypoxia-inducible factor 1
COX: Cytochrome c oxidase
OXPHOS: Oxidative phosphorylation
AG: Aerobic glycolysis
PDK1: Pyruvate dehydrogenase kinase-1
HKII: Hexokinase II
CaMKIIγ: Calcium/calmodulin-dependent protein kinase IIγ
FoxO1: Forkhead box O1
